# Dysregulated Glucuronidation of Bilirubin Exacerbates Liver Inflammation and Fibrosis in Schistosomiasis Japonica through the NF-κB Signaling Pathway

**DOI:** 10.3390/pathogens13040287

**Published:** 2024-03-28

**Authors:** Qingkai Xue, Yuyan Wang, Yiyun Liu, Haiyong Hua, Xiangyu Zhou, Yongliang Xu, Ying Zhang, Chunrong Xiong, Xinjian Liu, Kun Yang, Yuzheng Huang

**Affiliations:** 1 National Health Commission Key Laboratory of Parasitic Disease Control and Prevention, Jiangsu Provincial Key Laboratory on Parasite and Vector Control Technology, Jiangsu Institute of Parasitic Diseases, 117 Meiyuan Yangxiang, Wuxi 214064, China; xueqingkai@azyfy.com (Q.X.);; 2 Experimental Center of Clinical Research, The First Affiliated Hospital of Anhui University of Chinese Medicine, Hefei 230031, China; 3 Tropical Diseases Research Center, Nanjing Medical University, Wuxi 214064, China; 4 School of Public Health, Nanjing Medical University, Nanjing 211166, China; wangyuyan@jipd.com; 5 Department of Pathogen Biology, Key Laboratory of Antibody Techniques of National Health Commission, Nanjing Medical University, Nanjing 211166, China

**Keywords:** *Schistosoma japonicum*, hepatic fibrosis, UGT1A1, bilirubin, NF-κB

## Abstract

Hepatic fibrosis is an important pathological manifestation of chronic schistosome infection. Patients with advanced schistosomiasis show varying degrees of abnormalities in liver fibrosis indicators and bilirubin metabolism. However, the relationship between hepatic fibrosis in schistosomiasis and dysregulated bilirubin metabolism remains unclear. In this study, we observed a positive correlation between total bilirubin levels and the levels of ALT, AST, LN, and CIV in patients with advanced schistosomiasis. Additionally, we established mouse models at different time points following *S. japonicum* infection. As the infection time increased, liver fibrosis escalated, while liver UGT1A1 consistently exhibited a low expression, indicating impaired glucuronidation of bilirubin metabolism in mice. In vitro experiments suggested that SEA may be a key inhibitor of hepatic UGT1A1 expression after schistosome infection. Furthermore, a high concentration of bilirubin activated the NF-κB signaling pathway in L-O2 cells in vitro. These findings suggested that the dysregulated glucuronidation of bilirubin caused by *S. japonicum* infection may play a significant role in schistosomiasis liver fibrosis through the NF-κB signaling pathway.

## 1. Introduction

Schistosomiasis is a parasitic disease that is widely prevalent in tropical and subtropical regions, and it poses a serious health risk to humans [1,2]. The World Health Organization has estimated that approximately 779 million people are at risk of schistosomiasis infection worldwide, and more than 250 million people are infected with schistosomiasis [3,4]. The primary schistosomes affecting humans include *Schistosoma mansoni*, *Schistosoma japonicum* (*S. japonicum*), and *Schistosoma haematobium*, with *S. japonicum* being particularly prevalent in China [5,6]. *S. japonicum* infection primarily causes liver damage in the host [7], in which advanced schistosomiasis, a fibrosing portal hypertensive syndrome, develops after long-term repeated infection with schistosome cercariae, and the disease continues to progress in the absence of timely or complete anthelmintic treatment [8,9]. Complications such as liver fibrosis and ascites are significant contributors to mortality in patients with advanced schistosomiasis [10]. Serum biochemical tests have indicated that some patients with advanced schistosomiasis may exhibit abnormal changes in bilirubin metabolism, thus suggesting a potential absence of compensatory functions during liver fibrosis [11]. However, there is limited research on the association between alterations in bilirubin metabolism and liver fibrosis during schistosome infection.

The liver is an important metabolic organ of the body, containing metabolic enzymes that catalyze most phase I and phase II metabolic reactions and play important roles in the metabolism of endogenous and exogenous substances [12,13,14]. UDP-glucuronosyltransferases (UGTs), the important phase II metabolic enzymes in living organisms, are most actively expressed in the liver and catalyze glucuronidation reactions [15,16]. UGTs are regulated by various upstream nuclear receptors, including the pregnane X receptor (PXR) and the constitutional androstane receptor (CAR). Liver damage caused by schistosome infection is an important pathological manifestation of schistosomiasis [17]. In our previous study, through serum metabolomic analysis of *S. japonicum*-infected mice, we observed a decrease in the levels of serum glucuronic acid and its derivatives after schistosome infection [18]. We hypothesized that liver injury caused by *S. japonicum* infection might affect the glucuronidation reactions.

Bilirubin is a heme metabolism end product and is metabolized primarily by the liver [19]. The liver enzyme UGT1A1, one of the most important UGT isoforms, is the only enzyme that catalyzes the metabolic clearance of bilirubin. UGT1A1 is responsible for catalyzing the transfer of glucuronic acid from UDP-GlcUA to a receptor molecule (unconjugated bilirubin, UCB), resulting in the formation of a glucuronide conjugate of the receptor molecule (conjugated bilirubin, CB), which is subsequently excreted from the body [20,21]. Normally, moderate concentrations of bilirubin are considered antioxidant and beneficial to organisms [20,22]. However, abnormal bilirubin clearance in the body can trigger hyperbilirubinemia, thus leading to liver damage [23] and even irreversible brain damage [24,25]. Patients with liver disease and mutations in the UGT1A1 gene usually show abnormal bilirubin metabolism with hyperbilirubinemia [26]. Impaired bilirubin metabolism caused by *S. japonicum* infection may contribute to liver injury and fibrosis in schistosomiasis.

In this study, we investigated the impact of schistosome infection on bilirubin metabolism, building upon the identification of glucuronic acid metabolism in conventional mouse serum metabolomics and recognizing abnormal bilirubin metabolism in patients with advanced schistosomiasis. By analyzing the correlation between bilirubin metabolism and liver injury in patients with advanced schistosomiasis, we hypothesized that the high concentration of bilirubin might have an important role in liver fibrosis in schistosomiasis. Moreover, the mechanism of hepatocyte injury caused by the high bilirubin concentration was revealed through cellular experiments. This study provided new ideas for ameliorating the pathogenic mechanism of liver fibrosis in schistosomiasis by providing a new perspective on the relationship between liver metabolism and liver fibrosis.

## 2. Methods

### 2.1. Analysis of Blood Biochemical Examination Data for Patients with Advanced Schistosomiasis

Patients diagnosed with advanced schistosomiasis in Jiangsu Province underwent standardized assessments for advanced schistosomiasis status, and clinical blood biochemical examinations were conducted in accordance with ethical review number JIPD-2019-008. Inclusion criteria for cases were as follows: (i) The patients were identified and diagnosed as advanced schistosomiasis cases by rescue management organization of advanced schistosomiasis in Jiangsu Province. (ii) The cases are from areas where transmission of the disease was interrupted before 2000. Cases with failed audits and incomplete data were excluded. SPSS 22.0 was used for statistical analysis of the data. A Shapiro–Wilk normality test was conducted for each index. Normally distributed data variations are indicated with the mean ± standard deviation (SD). For data not following a normal distribution, variations are indicated by median and inter-quartile range (Q) (P25–P75). The correlation between total bilirubin (TBIL) and the four indicators of liver fibrosis were analyzed separately. Correlation analysis was performed with Pearson’s or Spearman’s correlation analysis, and positive rates were compared with χ^2^ tests with a test level of α = 0.05. A *p* value <0.05 indicates a statistically significant difference.

### 2.2. Cell Culture

LX-2 cells and L-O2 cells, previously stored in our laboratory, were utilized for this study. The cells were reconstituted from a tube in liquid nitrogen resuspended in DMEM (Hyclone, SH3002201, South Logan, OH, USA) containing 10% fetal bovine serum (Gibco, 10270106, Grand Island, NE, USA) and 1% penicillin-sulfur streptomycin, transferred to culture flasks, and incubated at 37 °C in an incubator containing 5% CO_2_. The cells were harvested when they reached 80% growth, and other cell experiments were performed.

### 2.3. Experimental Animals

Thirty-two female ICR mice (age: 6 weeks, weighing 20 ± 2 g) were purchased from Zhejiang Viton Lihua Experimental Animal Technology Co., Ltd. (Jiaxing, China) (license number: SCXK (Hangzhou, China) 2019-0001). The experimental animals were kept in the same environment at the Experimental Animal Center of Jiangsu Institute of Parasitic Diseases (license number: SYXK (Su) 2017-0050); ethical review number: JIPD-2020-002. Thirty-two mice were randomly divided into four groups of eight mice each: an uninfected group (control), 6-week infected group (6 w), 8-week infected group (8 w), and 10-week infected group (10 w). They were given free access to food and water and were acclimated for 1–2 weeks before infection. The mice in the infected group were percutaneously infected with 15 ± 2 cercariae per mouse [27]. The survival status of the mice was observed periodically.

### 2.4. Preparation of Cercariae

*S. japonicum* (Jiangsu strain) was preserved by the Jiangsu Institute of Parasitic Diseases. The cercariae released from infected Oncomelania snails in our laboratory were collected for animal experiments.

### 2.5. Soluble Egg Antigen (SEA) Preparation

*S. japonicum* eggs were collected from the livers and mesenteric venous plexus of New Zealand rabbits infected with cercariae at 42 d post-infection. The SEA was prepared according to the literature as follows [28]. The eggs were mixed with a PBS solution and ground for 20–30 min. After grinding, the supernatant was collected by centrifugation at 10,000× *g* for 10 min, and the process was repeated two or three times. The supernatant was filtered through a 0.22 μm pore-size filter membrane. The protein concentration was measured with a BCA protein quantification kit (Nanjing Vazyme Biotechnology, E112–01, Nanjing China) and stored at −80 °C for later use.

### 2.6. CCK-8 Method to Detect Cell Proliferation

LX-2 and L-O2 cells in the logarithmic growth phase were collected and counted and then seeded in 96-well plates at 2000 cells per well. Experimental wells contained different concentrations of SEA and bilirubin, whereas control wells contained cells and DMEM, and blank wells contained DMEM without cells. After incubation for 6–8 h, the medium in the experimental wells was replaced with DMEM containing different concentrations of SEA (0, 2.5, 5, 10, 20, or 40 μg/mL) and bilirubin (0, 20, 50, 100, or 200 μM, prepared as previously reported [29]). After treatment for 48 h and 16 h, respectively, 10 μL of CCK-8 reagent (Nanjing Vazyme Biotechnology, A311-01, Nanjing China) was added to each well, the cells were incubated, and the absorbance at 450 nm was measured with a microplate reader after 30 min, 1 h, 2 h, and 4 h. The best absorbance was selected, and the cell survival rate was calculated.

### 2.7. Detection of Apoptosis by Flow Cytometry

LX-2 and L-O2 cells in the logarithmic growth phase were collected, and cells were counted and seeded into six-well plates at 3 × 10^5^ cells per well. After cell attachment, the medium in each well was replaced with DMEM complete medium containing different concentrations of SEA (0, 5, 10, or 20 μg/mL), and subsequent flow cytometric assays were performed after 48 h of stimulation (Nanjing Vazyme Biotechnology, A211-01, Nanjing, China). Cells in 6-well plates were collected in flow tubes; cells were washed twice with precooled PBS; and supernatants were removed by centrifugation at 4 °C. Subsequently, 100 μL of 1× binding buffer was added, and cells were gently pipetted to form a single cell suspension. Then 5 μL Annexin V-FITC and 5 μL PI staining solution were added to each experimental-group tube, whereas untreated cell samples were used as a negative control. The experimental group tubes were stimulated with 20 μg/mL SEA for Annexin V-FITC and PI single staining for compensation adjustment, while gently blowing on the well; cells were incubated at room temperature in the dark for 10 min, and 400 μL 1× binding buffer was added and gently mixed. The stained samples were then detected by flow cytometry within 1 h.

### 2.8. ELISA for Serum Concentrations of Conjugated and Unconjugated Bilirubin

ELISA kits (Meimian, MM-0947M1, Nanjing, China; Meimian, MM-44711M1, Nanjing, China) and the samples to be tested were equilibrated to at room temperature for 20 min. To set up the standard wells and sample wells, we added 50 μL of each standard at different concentrations, or 10 μL of the sample to be tested plus 40 μL of the sample dilution buffer; no sample was added to the blank wells. To the blank, standard, and sample wells, we added 100 μL of horseradish peroxidase-labeled detection antibody, sealed the plate with sealing film, and incubated it for 60 min at 37 °C. The liquid was then discarded, and the plate was patted dry on blotting paper. Each well was filled with washing solution, which was allowed to stand for l min. The washing solution was then shaken off, and the plate was patted dry on blotting paper and washed five times. Subsequently, 50 μL each of substrates A and B was added to each well and incubated at 37 °C for 15 min in the dark. Then 50 μL of termination solution was added to each well, and the absorbance of each well was measured at 450 nm within 15 min. The bilirubin concentration of the corresponding sample was calculated according to the standard curve.

### 2.9. Histopathological Examination of Liver Samples

Small pieces of liver tissue from the right lobe in mice were fixed in a 4% paraformaldehyde solution for 24 h. After steps including gradient dehydration, transparency, wax immersion, embedding, sectioning, drying, dewaxing, rehydration, HE staining (Biosharp Biotechnology, BL700B, Hefei, China), and Masson staining (Solarbio Technology, G1340, Beijing, China), the sections were sealed, observed under a light microscope, and photographed.

### 2.10. The mRNA Expression Levels of the Genes Were Assessed Using Fluorescence Quantitative PCR

Total RNA from cells or tissues was extracted with RNA-easy Isolation Reagent (Nanjing Vazyme Biotechnology, R701, Nanjing China), and an RNA purity (OD_260_/OD_280_ ratio in the range of 1.8–2.2) was verified with a NanoDrop 2000 instrument. The cDNA was obtained through reverse transcription with a kit (Nanjing Vazyme Biotechnology, R323-01, Nanjing China). RNA levels were assessed with a LightCycler 480 instrument with SYBR qPCR Master Mix (Nanjing Vazyme Biotechnology, Q711-02, Nanjing China) and the indicated primers (Table S1). After the threshold cycle (Ct) was obtained, the relative expression of the target gene was calculated with the 2^−ΔΔCt^ method with β-actin as the internal reference.

The primers were synthesized by Shanghai Bioengineering Co., Ltd. (Shanghai, China), and the relevant primer sequences are shown in Table S1.

### 2.11. Western Blot Assays

The total protein from cells or liver tissues was extracted, and the protein concentration was detected with the BCA method. Protein samples were mixed with 6× SDS protein loading buffer at a ratio of 5:1 and boiled for 10 min at 95–100 °C. Each well was loaded with 30 μg of protein and subjected to SDS-PAGE gel electrophoresis. The protein was transferred to a PVDF membrane, which was blocked with 5% skim milk powder for 1–2 h. Primary antibodies (specific for UGT1A1, Proteintech, 23495-1-AP, Wuhan, China; p65, Abcam, ab16502, Cambridge, UK; IκB-α, Abcam, ab32518, Cambridge, UK; IKK-β, Abcam, ab124957, Cambridge, UK; p-IκB-α, Abcam, ab133462, Cambridge, UK; p-IKK-β, Affinity, AF3013,Nanjing, China; p-p65, Cell Signaling, 3033T, Boston, USA; GAPDH, Absin, abs132004, Shanghai, China; and β-actin, Biosharp, BL005, Hefei, China) were added and incubated overnight at 4 °C. The blots were then washed three times for 10 min with TBST. Secondary antibodies were added and incubated for 1–2 h on a shaker at room temperature. After extensive washing of the membrane, protein bands were visualized with a Bio-Rad ChemiDoc XRS+ imaging system and quantified through densitometry in Image J V. 1.52 software.

### 2.12. Data Processing and Statistical Analysis

Variations in the experimental data are indicated as mean ± SD. SPSS 22.0 was used for statistical analysis, and one-way ANOVA and a Dunnett-t test were used to compare the significance of differences in data among multiple groups with a significance level of α = 0.05. A *p* value <0.05 indicated a statistically significant difference (* *p* < 0.05, ** *p* < 0.01, *** *p* < 0.001, **** *p* < 0.0001). Data visualization was performed with GraphPad Prism 9.0.

## 3. Results

### 3.1. Changes and Correlation Analysis of Total Bilirubin and Indicators of Liver Injury and Liver Fibrosis in Patients with Advanced Schistosomiasis

Previous studies have reported bilirubin decompensation in patients with advanced schistosomiasis. To comprehensively understand this phenomenon, a detailed analysis was conducted using data from blood biochemical examinations conducted on a cohort of 745 patients with advanced schistosomiasis in Jiangsu Province. This analysis focused on an array of pertinent indicators encompassing liver injury indicators (ALT and AST), liver fibrosis markers (HA, LN, PIIIP, and CIV), and the total bilirubin levels detected within the patient’s bloodstream. A Shapiro–Wilk normality test was conducted for each indicator, showing that all data did not follow a normal distribution. The median levels of TBIL, ALT, AST, HA, LN, PIIIP, and CIV were 12.8 μmol/L, 14.2 U/L, 29.00 U/L, 154.59 ng/mL, 68.71 ng/mL, 14.15 ng/mL, and 25.14 ng/mL, respectively. In addition, the positivity rates for TBIL, ALT, AST, HA, LN, PIIIP, and CIV in the population were 24.16%, 4.97%, 16.51%, 66.71%, 59.46%, 11.95%, and 41.74%, respectively (Table 1). Patients with abnormal TBIL also had significant abnormalities in blood indicators, such as AST, LN, and CIV (all *p* < 0.001) (Tables S2–S7). Spearman’s correlation analysis was performed between TBIL and liver injury and liver fibrosis-associated indicators (ALT, AST, HA, LN, PIIIP, and CIV) separately. TBIL was positively correlated with AST, ALT, LN, and CIV (all *p* < 0.001) (Table 2). These findings suggest a connection between impaired bilirubin metabolism and liver fibrosis in patients with advanced schistosomiasis.

### 3.2. SEA Inhibits LX-2 and L-O2 Cell Proliferation and Promotes Apoptosis In Vitro

**Figure 1:**
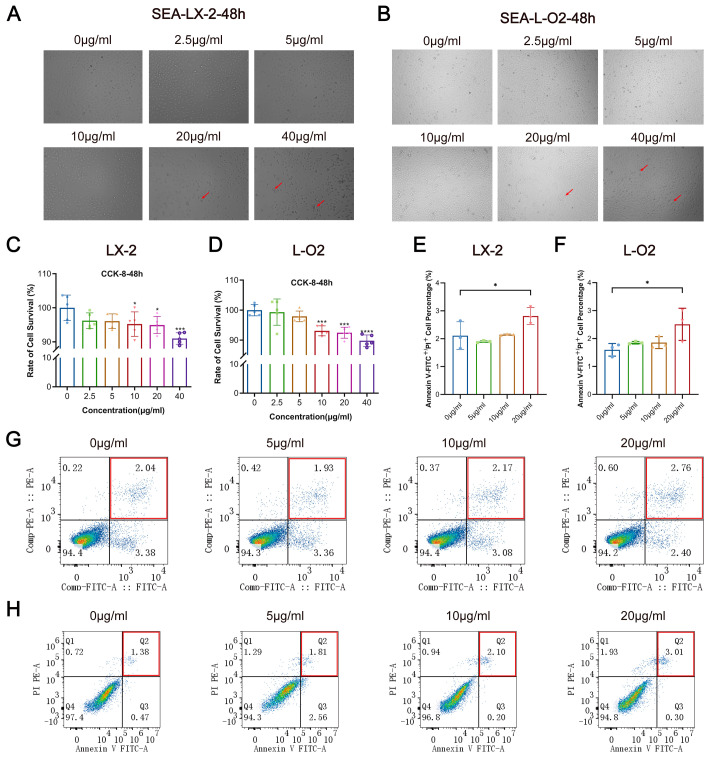
Regulatory effect of SEA on LX-2 and L-O2 cells in vitro. (**A**) Changes in cell morphology of LX-2 cells stimulated by SEA at different concentrations for 48 h in vitro (20×). (**B**) Changes in cell morphology of L-O2 cells stimulated by SEA at different concentrations for 48 h in vitro (20×). (**C**) Changes in cell proliferation of LX-2 cells stimulated by SEA at different concentrations for 48 h in vitro. (**D**) Changes in cell proliferation of L-O2 cells stimulated by SEA at different concentrations for 48 h in vitro. (**E**) Statistical analysis of cell apoptosis in LX-2 cells after 48 h stimulation by SEA at different concentrations in vitro. (**F**) Statistical analysis of cell apoptosis in L-O2 cells after 48 h stimulation by SEA at different concentrations in vitro. (**G**) Flow cytometry detection in LX-2 cells after 48 h stimulation by SEA at different concentrations in vitro. (**H**) Flow cytometry detection in L-O2 cells after 48 h stimulation by SEA at different concentrations in vitro. At least three replicate samples were used for each experiment. Statistical significance is shown as * *p* < 0.05, *** *p* < 0.001, **** *p* < 0.0001.

SEA plays a pivotal role as a pathogenic factor in schistosomiasis-related liver injury. It induces damage to liver cells, triggers an immune-inflammatory response, and subsequently activates liver stellate cells, ultimately resulting in liver fibrosis. Therefore, we studied the effects of SEA on LX-2 and L-O2 cells in vitro. LX-2 and L-O2 cells were treated with different concentrations of SEA in vitro, and cell morphology, proliferation, and apoptosis were observed. As the SEA concentration increased, the cell morphology underwent varying degrees of alteration. Particularly in the high concentration group (40 μg/mL), some cells exhibited distinct changes, such as wrinkling, rounding, and poor definition (Figure 1A,B). SEA significantly inhibited the proliferation of both LX-2 and L-O2 cells in vitro (*p* < 0.01, *p* < 0.0001) (Figure 1C,D). Apoptosis was assessed using flow cytometry, where Annexin V-FITC^+^/PI^+^ indicated apoptotic and necrotic cells. The grouping for apoptosis assays was based on the results of the cell proliferation assay, and intermediate SEA concentrations (5, 10, and 20 μg/mL) were selected to stimulate LX-2 and L-O2 cells in vitro. SEA caused apoptosis in LX-2 cells in vitro (*p* < 0.05) (Figure 1E,G), whereas the percentage of Annexin V-FITC^+^/PI^+^ cells was 2.11 ± 0.41% in the control group and 2.81 ± 0.25% in the 20 μg/mL concentration group. Similarly, SEA induced apoptosis in L-O2 cells in vitro (*p* < 0.05) (Figure 1F,H). In the control group, the percentage of Annexin V-FITC^+^/PI^+^ cells was 1.60 ± 0.19%, and in the 20 μg/mL concentration group, it increased to 2.51 ± 0.47%.

### 3.3. SEA Inhibits the mRNA Expression of UGT1A1 in LX-2 Cells and L-O2 Cells In Vitro

**Figure 2:**
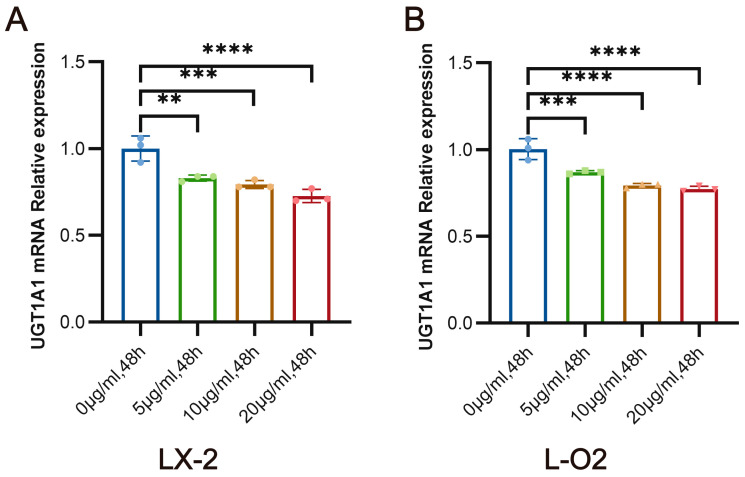
Effects of SEA treatment on the mRNA expression of UGT1A1 in L-O2 and LX-2 cells in vitro. (**A**) mRNA expression of UGT1A1 in LX-2 cells stimulated by different concentrations of SEA for 48 h in vitro. (**B**) mRNA expression of UGT1A1 in L-O2 cells stimulated by different concentrations of SEA for 48 h in vitro. At least three replicate samples were used for each experiment. Statistical significance is shown as ** *p* < 0.01, *** *p* < 0.001, **** *p* < 0.0001.

In our previous study, we found through metabolomics studies that schistosome infection can affect liver glucuronidation. UGT1A1, as one of the most important metabolic enzymes that catalyze liver glucuronidation, may be affected in different degrees during schistosome infection. We observed the effects of different concentrations of SEA (0 μg/mL, 5 μg/mL, 10 μg/mL, and 20 μg/mL) on the expression of the UGT1A1 gene in L-O2 cells and LX-2 cells. SEA treatment significantly inhibited the mRNA expression of UGT1A1 in LX-2 cells after SEA treatment for 48 h (*p* < 0.01), as compared with control group levels (Figure 2A). Similarly, compared with the control group, mRNA expression of UGT1A1 in L-O2 cells was significantly inhibited 48 h after SEA treatment (*p* < 0.0001) (Figure 2B). These findings suggest that SEA may play a crucial role in inducing the suppression of hepatic UGT1A1 expression after *S. japonicum* infection.

### 3.4. S. japonicum Infection Suppresses Hepatic UGT1A1 Expression, Thereby Impairing Glucuronidation of Bilirubin

**Figure 3:**
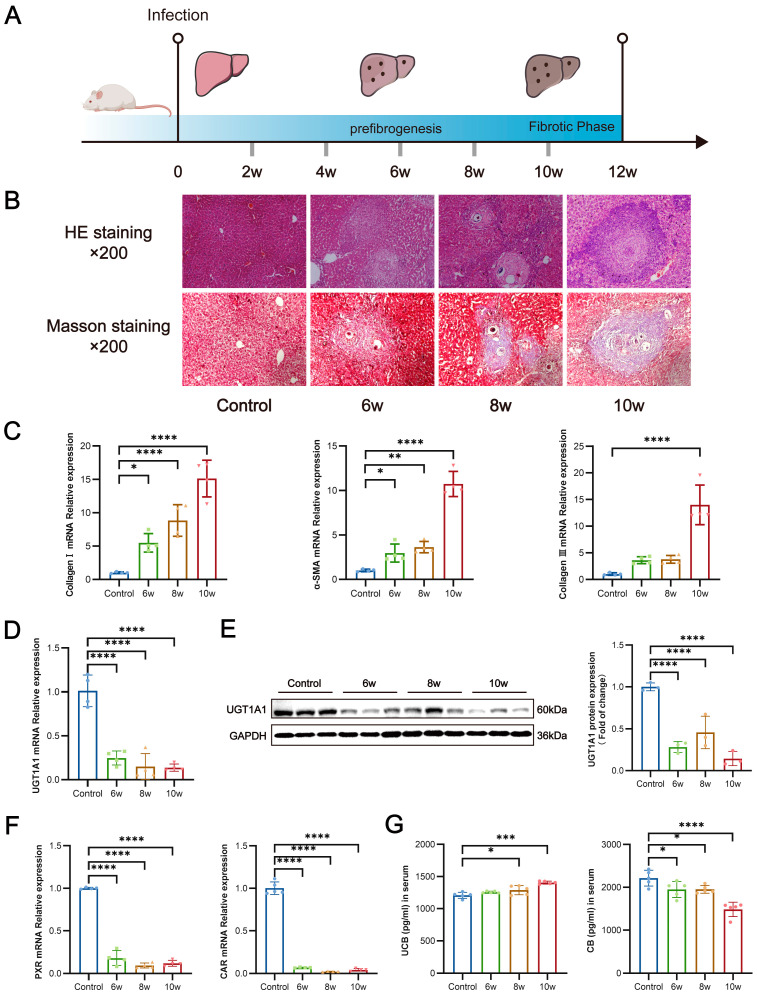
Relationship between dysregulated glucuronidation of bilirubin and liver fibrosis in *S. japonicum*-infected mice. (**A**) Schematic diagram of the experimental design of the mouse model infected with *S. japonicum*. (**B**) HE and Masson staining results of liver pathology (200×). (**C**) mRNA expression of α-SMA, collagen I, and collagen III in the liver of mice. (**D**) mRNA expression of UGT1A1 in the liver of mice. (**E**) Measurement of UGT1A1 protein levels in the liver of mice by Western blotting (normalized to GAPDH). (**F**) The mRNA expression of PXR and CAR in the liver of mice. (**G**) Serum levels of unconjugated bilirubin and conjugated bilirubin in mice. At least three replicate samples were used for each experiment. Statistical significance is shown as * *p* < 0.05, ** *p* < 0.01, *** *p* < 0.001, **** *p* < 0.0001.

After *S. japonicum* infection, the SEA released from the eggs can induce the formation of egg granulomas and the secondary formation of liver fibrosis. The hepatic pathological changes were observed in mouse models of different times (6, 8, and 10 weeks) after *S. japonicum* infection (Figure 3A). HE staining showed the egg granulomas of the liver, and Masson staining showed deposition of collagen fibers in the liver. HE staining indicated that the egg granulomas started to form approximately 6 weeks after schistosome infection, and the granuloma area of the eggs in the liver increased with infection time (Figure 3B). Masson staining indicated that the collagen fibers began to deposit around 6 weeks after infection, fibrosis started to form, and the collagen fibers in the liver showed substantial deposition at 10 weeks after infection. Fibrosis also began to develop (Figure 3B). The expression of liver-associated liver fibrosis factors (collagen I, collagen III, and α-SMA) in *S. japonicum*-infected mice, detected by qRT-PCR, reflected the progression of liver fibrosis. The expression of liver fibrosis indicators in mice increased with time after infection. Compared with that in the control group, the expression of mRNA of collagen I and α-SMA in the liver in mice 6, 8, and 10 weeks after infection had significantly increased (*p* < 0.0001, *p* < 0.0001), and the expression of collagen III had significantly increased at 10 weeks after infection (*p* < 0.0001) (Figure 3C). Chronic stimulation by eggs deposited in the liver after schistosome infection led to liver fibrosis, and the liver fibrosis damage gradually increased with time, in agreement with the results of Masson staining for liver pathology.

SEA inhibited the expression of UGT1A1 in liver-associated cells in vitro, whereas the changes in hepatic UGT1A1 expression and its catalyzed bilirubin metabolism in *S. japonicum*-infected states in vivo were unclear. Therefore, we conducted qRT-PCR to detect the expression of UGT1A1 in the liver of mice at different times after *S. japonicum* infection. The mRNA expression of UGT1A1 in the liver of mice at 6, 8, and 10 weeks after infection was consistently lower than that in the control group (*p* < 0.0001) (Figure 3D). Western blotting detection of the expression of UGT1A1 protein yielded results consistent with the qRT-PCR results (*p* < 0.0001) (Figure 3E). We also detected the mRNA expression of PXR and CAR, crucial transcriptional regulators upstream of UGT1A1, by qRT-PCR. Compared with that in the control group, the mRNA expression of PXR and CAR in the liver at 6, 8, and 10 weeks after infection was significantly lower, and the changing trend was the same as that of UGT1A1 (*p* < 0.0001, *p* < 0.0001) (Figure 3F). The metabolism of bilirubin is catalyzed by UGT1A1 in the liver. The levels of conjugated and unconjugated bilirubin in the serum in schistosome-infected mice were measured by ELISA. The serum levels of conjugated bilirubin in mice showed a significant decrease (*p* < 0.0001), whereas those of unconjugated bilirubin showed a significant increase (*p* < 0.0001) with increasing time after infection (Figure 3G). This evidence suggested that bilirubin metabolism is impaired in mice after *S. japonicum* infection, consistent with the inhibition of hepatic UGT1A1 expression following infection.

### 3.5. Bilirubin Inhibits the Proliferation of L-O2 Cells While Activating the NF-κB Signaling Pathway In Vitro

**Figure 4:**
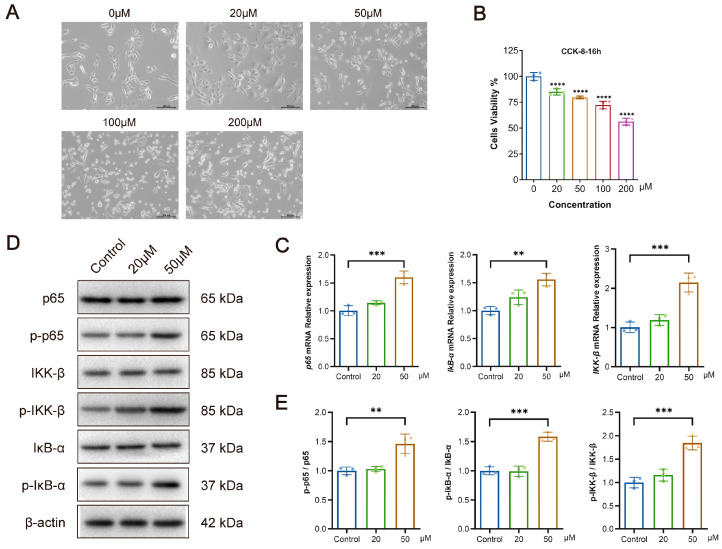
Effects of bilirubin stimulation in vitro on L-O2 cells. (**A**) Effects of bilirubin on L-O2 cell morphology (100×). (**B**) CCK-8 assay of bilirubin-treated L-O2 cells. (**C**) mRNA expression of p65, IκB-α, and IKK-β in L-O2 cells stimulated by bilirubin. (**D**) Measurement of p65, p-p65, IKK-β, p-IKK-β, IκB-α, and p-IκB-α protein levels in L-O2 cells by Western blotting. (**E**) Statistical analysis of p-p65, p-IKK-β, and p-IκB-α protein levels in L-O2 cells (normalized to p65, IKK-β, or IκB-α). At least three replicate samples were used for each experiment. Statistical significance is shown as ** *p* < 0.01, *** *p* < 0.001, **** *p* < 0.0001.

**Figure 5:**
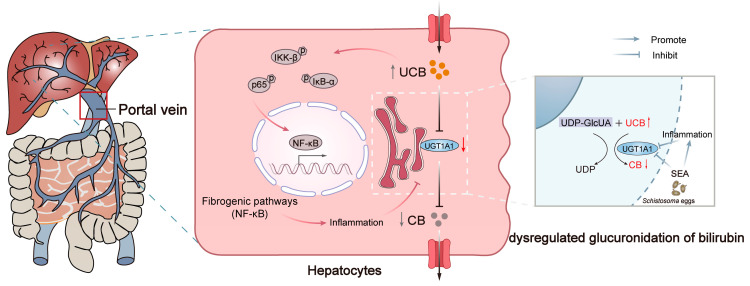
Dysregulated glucuronidation of bilirubin exacerbates liver inflammation and fibrosis in schistosomiasis japonica through the NF-κB signaling pathway.

Impaired bilirubin metabolism may cause hyperbilirubinemia, and excessive bilirubin levels may increase the risk of cytotoxicity. To investigate the relationship between bilirubin and liver injury, we next directly treated L-O2 cells with various concentrations of bilirubin in vitro to investigate its effects. The low concentration (20 μM) of bilirubin had no clear effect on the morphology of L-O2 cells, whereas the high concentration (>50 μM) of bilirubin resulted in a round, wrinkled appearance, and other morphological changes (Figure 4A). Meanwhile, CCK-8 assays indicated that a high concentration of bilirubin inhibited the proliferation of L-O2 cells (*p* < 0.0001) (Figure 4B). The effects of bilirubin treatment on the NF-κB-associated signaling pathway in L-O2 cells were detected with qRT-PCR, which indicated that 50 μM of bilirubin significantly upregulated the mRNA expression of p65, IκB-α, and IKK-β (*p* < 0.001, *p* < 0.01, *p* < 0.001) (Figure 4C). Western blotting was performed to detect the expression of related phosphorylated proteins and also confirmed that the expression of p65, IκB-α, and IKK-β protein phosphorylation levels significantly increased after bilirubin treatment in L-O2 cells (*p* < 0.01, *p* < 0.001, and *p* < 0.001, respectively) (Figure 4D,E). These findings suggest that excessive bilirubin can cause liver injury and activate NF-κB, the core signaling pathway in liver fibrosis. Therefore, the inhibition of bilirubin metabolism due to dysregulated glucuronidation reactions after *S. japonicum* infection may exacerbate schistosomiasis liver fibrosis by activating the NF-κB signaling pathway (Figure 5).

## 4. Discussion and Conclusions

Emerging evidence suggests that alterations in metabolism are not only a feature of but also may play a significant role in the pathogenesis of fibrosis. Recent studies have highlighted increased glycolysis and glutamine metabolism as modulators of fibrosis, with a particular emphasis on their potential application in novel therapeutic approaches. Therefore, analyzing metabolic changes in hepatic disease states is valuable for comprehending the disease process [30]. In this study, we focused on the relationship between the glucuronidation metabolism of the endogenous substance bilirubin and schistosomiasis liver fibrosis. The objective was to uncover potential mechanisms of metabolic disorders participating in the process of schistosomiasis liver fibrosis.

Schistosomiasis liver fibrosis is an important clinical manifestation of the chronic development of schistosomiasis and an important complication leading to death in patients with advanced schistosomiasiss [31]. The four indicators of liver fibrosis (HA, LN, PIIIP, and CIV) in the serum have important application value in the diagnosis of schistosomiasis fibrosis. Analysis of clinical biochemical examination findings for existing patients with advanced schistosomiasis in Jiangsu Province indicated that all patients had abnormalities in the four indicators of liver fibrosis to different degrees. We simulated the development process of schistosomiasis liver fibrosis by establishing mouse models of different times after *S. japonicum* infection and evaluated the severity of liver fibrosis. Liver damage in mice increased after *S. japonicum* infection, as did the symptoms of liver fibrosis. Previous studies have generally concluded that liver fibrosis in schistosomiasis was due primarily to SEA stimulation of hepatic stellate cells, thereby eliciting an immune response leading to excessive deposition of extracellular matrix and ultimately liver fibrosis [32]. We also showed that SEA inhibited LX-2 and L-O2 cell proliferation in vivo and promoted apoptosis in vitro, thus suggesting that SEA may be an important inducing factor promoting liver injury in schistosome infection.

With the rise in the application of metabolomics, the link between liver metabolism and liver fibrosis injury has received increasing attention [33,34,35]. Previously, through metabolomics, we screened and identified glucuronic acid metabolism as a distinct metabolic pathway linked to schistosomiasis infection. UGT1A1 utilizes UDP-GlcUA, a significant product generated in glucuronic acid metabolism, as a co-substrate to catalyze the glucuronidation reaction, and it is also associated with the progression of multiple diseases [36,37,38]. In addition, there are two main causes of hepatotoxicity caused by low activity of the UGT1A1 gene: drug-induced and bilirubin-induced [39]. The UGT1A1 enzyme is the only enzyme known to catalyze bilirubin metabolism in the body, and patients with advanced schistosomiasis show varying degrees of abnormal bilirubin metabolism. The biological role of the UGT1A1 enzyme in endogenous bilirubin metabolism in the body has been well established in the past decades [40], and changes in UGT1A1 expression in liver disease states have been studied. In liver injury caused by *Schistosoma mansoni* infection, the expression of hepatic UGTs did not change significantly in the early stages of *Schistosoma mansoni* infection, whereas the mRNA expression of hepatic UGT1A1 decreased in the chronic infection period [41,42]. Although the pathogenesis of *S. japonicum* and *S. mansoni* is similar, in our study, the mRNA expression of hepatic UGT1A1 was consistently low from the beginning of the granuloma formation of *S. japonicum* eggs (6 weeks after infection) to the development of chronic fibrosis (10 weeks after infection), as also verified by Western blotting. A low expression of UGT1A1 inhibited bilirubin metabolism in mice, thus manifesting as increased serum unconjugated bilirubin levels and decreased conjugated bilirubin levels. Notably, SEA inhibited the mRNA expression of UGT1A1 in LX-2 cells and L-O2 cells, thus suggesting that SEA may be an important inducing factor inhibiting the expression of UGT1A1 in the liver. On the basis of the above results, we elucidated the effect of schistosome infection on bilirubin metabolism.

Schistosome egg granulomas are the basis for the formation of liver fibrosis and result from an inflammatory response due to continual stimulation by the egg antigen, which usually accompanies the entire process of liver fibrosis [43]. We found that the formation of schistosome egg granulomas and liver fibrosis appeared to temporally coincide with the inhibition of hepatic UGT1A1 expression. The activation of inflammation-associated signaling factors has been suggested to inhibit the expression of UGT1A1 [44]. The inhibition of hepatic UGT1A1 expression caused by *S. japonicum* infection may be induced directly by the action of SEA on hepatocytes on the one hand, and indirectly through the SEA-induced inflammatory response in the liver on the other hand. In addition, several transcriptional regulators in vivo play important roles in hepatic UGT1A1 expression, such as PXR, CAR, FXR, Nrf2, and AhR. Our animal experiments showed that the decrease in hepatic UGT1A1 expression caused by *S. japonicum* infection was accompanied by the suppression of PXR and CAR expression. A combination of factors mediates the suppression of hepatic UGT1A1 expression after schistosome infection and further causes impaired metabolism of endogenous substances, and the hepatic accumulation of substances can lead to hepatocyte toxicity and aggravate liver injury. In *S. japonicum*, liver fibrosis formation is the result of a vicious cycle formed by two pathways, the hepatic inflammatory response and metabolic disorders, which may play important roles in promoting each other during the development of liver fibrosis. Moreover, by activating the expression of target genes such as PXR, CAR, and FXR in individuals with low UGT1A1 expression, the expression of UGT1A1 has been suggested to be upregulated and to counteract liver injury [45]. A variety of clinical drugs are used to increase the expression of UGT1A1 by acting on the above regulatory pathways to achieve hepatoprotective effects [46,47,48], thus showing that increasing the expression of liver-associated metabolic enzyme UGT1A1 has an important role in the treatment of related liver diseases. Therefore, increasing hepatic UGT1A1 expression to promote substance metabolism may be important in alleviating *S. japonicum* liver fibrosis.

Elevated bilirubin in patients with liver disease has long been considered a serological indicator of disease severity. In our study, by correlating TBIL abnormalities with indicators of liver fibrosis in patients with advanced schistosomiasis, we found correlations among TBIL and ALT, AST, LN, and CIV, as well as significant differences in AST, LN, and CIV levels in the blood of patients with impaired bilirubin metabolism, thus indicating that abnormal bilirubin metabolism correlates with schistosomiasis liver fibrosis. On the one hand, alterations in metabolic substrates can disrupt the normal physiological function of hepatocytes; on the other hand, it can also exert an immunomodulatory effect. It has been suggested that physiological or moderate concentrations of bilirubin can exert negative immunomodulatory effects by inhibiting the NF-κB signaling pathway [49]. In contrast, our in vitro study did not reveal a significant effect of moderate concentrations of bilirubin on the NF-κB signaling pathway in hepatocytes. However, intriguingly, our data suggest that high concentrations of bilirubin activate the NF-κB signaling pathway in hepatocytes, corroborating a previous study’s perspective [26], which also suggests that bilirubin may play a role in bi-directional immune regulation within the body. The NF-κB signaling pathway, the core signaling hub of hepatocytes, integrates the activity of various stress-associated and inflammatory mediators and is also a central pathway in schistosomiasis liver fibrosis [50,51]. The excessive accumulation of unconjugated bilirubin in the liver because of the low expression of hepatic UGT1A1 caused by *S. japonicum* infection may further promote the progression of *S. japonicum* liver fibrosis by activating the NF-κB signaling pathway.

In conclusion, we provided evidence of the underlying impaired bilirubin metabolism in patients with schistosomiasis and revealed the relationship between pathogenic infection-induced liver injury and dysregulated glucuronidation of bilirubin due to inhibition of hepatic metabolizing enzyme (UGT1A1) expression. We additionally investigated the effects of impaired hepatic metabolism on liver fibrosis in schistosomiasis from the perspective of liver metabolism. This study also provided a foundation for further investigation of hepatic glucuronic acid metabolism in the pathogenesis of liver fibrosis in schistosomiasis.

## Figures and Tables

**Table 1:** Test results for blood total bilirubin, liver injury, and liver fibrosis indicators in patients with advanced schistosomiasis in Jiangsu Province.

	TBIL (2–20 μmol/L)	ALT (5–45 U/L)	AST (5–45 U/L)	HA (<100 ng/mL)	LN (<50 ng/mL)	PIIIP (<30 ng/mL)	CIV (<30 ng/mL)
Median	12.8	14.2	29	154.59	68.71	14.15	25.14
Upper quartile	8.65	8	22.05	82.39	38.28	7.15	17.36
Lower quartile	19.9	24	38.08	304.83	157.92	23.00	48.33
Number of positives	180	37	123	497	443	89	311
Total number of tests	745	745	745	745	745	745	745
Positivity rate	24.16%	4.97%	16.51%	66.71%	59.46%	11.95%	41.74%

**Table 2:** Analyzing the correlation between TBIL and indicators of liver injury and fibrosis.

	ALT	AST	LN	HA	PIIIP	CIV
r	0.433	0.416	0.349	0.023	−0.277	0.311
*p*	<0.001	<0.001	<0.001	0.528	<0.001	<0.001

## Data Availability

All data supporting the conclusions of this study are included in the article.

## Supplementary Materials

The following supporting information can be downloaded at: https://www.mdpi.com/article/10.3390/pathogens13040287/s1. Table S1. Sequences of all primers analyzed by real-time PCR; Table S2. Comparison of ALT positive rate in patients with normal and abnormal TBIL; Table S3. Comparison of AST positive rate in patients with normal and abnormal TBIL; Table S4. Comparison of HA positive rate in patients with normal and abnormal TBIL; Table S5. Comparison of LN positive rate in patients with normal and abnormal TBIL; Table S6. Comparison of PIIIP positive rate in patients with normal and abnormal TBIL; Table S7. Comparison of CIV positive rate in patients with normal and abnormal TBIL.

## Abbreviations

UGT1A1: UDP-glucuronosyltransferases 1A1; TBIL: Total bilirubin; ALT: Alanine aminotransferase; AST: Aspartate aminotransferase; HA: Hyaluronic acid; LN: Laminin; CIV: Type IV Collagen; PIIINP: Type III procollagen; SEA: Soluble egg antigen; UCB: Unconjugated bilirubin; CB: Conjugated bilirubin; PXR: Pregnane X receptor; CAR: Constitutive androstane receptor; Collagen I: Type I collagen; Collagen III: Type III collagen; α-SMA: Alpha-smooth muscle actin; NF-κB: Nuclear factor kappa-B; IκB: Inhibitory protein of NF-κB; IKK: IκB kinase; qRT-PCR: Quantitative real-time PCR; ELISA: Enzyme-linked immunosorbent assay; HE: Hematoxylin-eosin; FXR: Farnesoid X receptor; Nrf2: Nuclear factor erythroid-2-related factor 2; AhR: Aryl hydrocarbon receptor.
