# Supplementary material for: Dysregulated Glucuronidation of Bilirubin Exacerbates Liver Inflammation and Fibrosis in Schistosomiasis Japonica through the NF-κB Signaling Pathway
Source: Pathogens. 2024 Mar 28;13(4):287. doi: 10.3390/pathogens13040287 (PMC11054532; doi:10.3390/pathogens13040287)
Supplement: Supplementary file 1 [file pathogens-13-00287-s001.zip › pathogens-2872041-supplementary.pdf]

Table S1. Sequences of all primers analyzed by real-time PCR

	Gene	Forward (5′ → 3′)	Reverse (5′ → 3′)
Human	UGT1A1	CTCTCCAGCCTTCACAAG	CGGTAGCCATAAGCACAA
	p65	GGCAGGAAGAGGAGGTTTCG	AGGACGTTGTGTTCCTTCCG
	IKK-β	TCCTTCAGGACCGAGCAAAG	CGTTCGATGATGCGGTTGTT
	IκB-α	AGAAGGAGCGGCTACTGGAT	GCCTCCGCCACTTACGAG
	β-actin	GGCACTCTTCCAGCCTTCC	GAGCCGCCGATCCACAC
Mouse	UGT1A1	CCTATGTCAACGCCTCTG	CATCATCACCATCGGAACT
	PXR	CCCATCAACGTAGAGGAGGA	TCTGAAAAACCCCTTGCATC
	CAR	CTCAAGGAAAGCAGGGTCAG	AGTTCCTCGGCCCATATTCT
	Collagen I	ACTGGCAACCTCAAGAAGTCCC	ACGCAAGGCCATGAGACTCC
	Collagen III	ACGCAAGGCCATGAGACTCC	GCAAACAGGGCCAATGTCCA
	α-SMA	CTATGCTCTGCCTCATGCCA	CTCACGCTCAGCAGTAGTCA
	β-actin	CCACCATGTACCCAGGCATT	ACGCAGCTCAGTAACAGTCC

Table S2. Comparison of ALT positive rate in patients with normal and abnormal TBIL

TBIL	ALT		Total number	χ^2	*P*
	Positive	Negative			
Positive	14	166	180	3.975	0.046
Negative	23	542	565		
Total number	37	708	745		

Table S3. Comparison of AST positive rate in patients with normal and abnormal TBIL

TBIL	AST		Total number	χ^2	*P*
	Positive	Negative			
Positive	57	123	180	39.555	0.000
Negative	66	499	565		
Total number	123	622	745		

Table S4. Comparison of HA positive rate in patients with normal and abnormal TBIL

TBIL	HA		Total number	χ^2	*P*
	Positive	Negative			
Positive	124	56	180	0.507	0.477
Negative	373	192	565		
Total number	497	248	745		

Table S5. Comparison of LN positive rate in patients with normal and abnormal TBIL

TBIL	LN		Total number	χ^2	*P*
	Positive	Negative			
Positive	134	46	180	22.100	0.000
Negative	309	256	565		
Total number	443	302	745		

Table S6. Comparison of PIIIP positive rate in patients with normal and abnormal TBIL

TBIL	PIIIP		Total number	χ^2	*P*
	Positive	Negative			
Positive	18	162	180	0.855	0.355
Negative	71	494	565		
Total number	89	656	745		

Table S7. Comparison of CIV positive rate in patients with normal and abnormal TBIL

TBIL	CIV		Total number	χ^2	*P*
	Positive	Negative			
Positive	109	71	180	34.012	0.000
Negative	203	362	565		
Total number	312	433	745		
